# Genome-wide identification of allele-specific expression (ASE) in response to Marek’s disease virus infection using next generation sequencing

**DOI:** 10.1186/1753-6561-5-S4-S14

**Published:** 2011-06-03

**Authors:** Sean MacEachern, William M Muir, Seth Crosby, Hans H Cheng

**Affiliations:** 1 USDA, ARS, Avian Disease and Oncology Laboratory (ADOL), East Lansing, MI 48823, USA; 2 Current address: Cobb Vantress Inc., Siloam Springs, AR 72761, USA; 3 Department of Animal Sciences, Purdue University, West Lafayette, IN 47907, USA; 4 Genome Sequencing Center, Washington University in St. Louis, St. Louis, MO 63108, USA

## Abstract

**Background:**

Marek’s disease (MD), a T cell lymphoma induced by the highly oncogenic α-herpesvirus Marek’s disease virus (MDV), is the main chronic infectious disease concern threatening the poultry industry. Enhancing genetic resistance to MD in commercial poultry is an attractive method to augment MD vaccines, which is currently the control method of choice. In order to optimally implement this control strategy through marker-assisted selection (MAS) and to gain biological information, it is necessary to identify specific genes that influence MD incidence.

**Methods:**

A genome-wide screen for allele-specific expression (ASE) in response to MDV infection was conducted. The highly inbred ADOL chicken lines 6 (MD resistant) and 7 (MD susceptible) were inter-mated in reciprocal crosses and half of the progeny challenged with MDV. Splenic RNA pools at a single time after infection for each treatment group point were generated, sequenced using a next generation sequencer, then analyzed for allele-specific expression (ASE). To validate and extend the results, Illumina GoldenGate assays for selected cSNPs were developed and used on all RNA samples from all 6 time points following MDV challenge.

**Results:**

RNA sequencing resulted in 11-13+ million mappable reads per treatment group, 1.7+ Gb total sequence, and 22,655 high-confidence cSNPs. Analysis of these cSNPs revealed that 5360 cSNPs in 3773 genes exhibited statistically significant allelic imbalance. Of the 1536 GoldenGate assays, 1465 were successfully scored with all but 19 exhibiting evidence for allelic imbalance.

**Conclusions:**

ASE is an efficient method to identify potentially all or most of the genes influencing this complex trait. The identified cSNPs can be further evaluated in resource populations to determine their allelic direction and size of effect on genetic resistance to MD as well as being directly implemented in genomic selection programs. The described method, although demonstrated in inbred chicken lines, is applicable to all traits in any diploid species, and should prove to be a simple method to identify the majority of genes controlling any complex trait.

## Background

The comprehensive identification of genes underlying phenotypic variation of complex traits such as disease resistance remains one of the greatest challenges in biology despite having genome sequences and more powerful tools. Most genome-wide screens lack sufficient resolving power as they typically depend on linkage. An alternate method is to screen for allele-specific expression (ASE), a simple yet powerful approach, where the expression of each gene allele is compared **within** an RNA sample. When the expression of the alleles is not equal, then one can **unequivocally** declare ASE and the presence of a polymorphic cis-acting (genetic) element for that gene as linkage disequilibrium (LD) is confined to the transcriptional unit. The only requirements for ASE to work are: (1) the assumption that variations in expression between alleles of a gene are responsible for part of the phenotypic variation, and (2) the existence of a cSNP to monitor the alleles. In this proof-of-concept study, a genome-wide screen for ASE in response to MDV infection in chicken was conducted using next generation sequencing to identify cSNPs and preliminary evidence for allelic imbalance followed by validation with Illumina Illumina GoldenGate assays to validate.

## Methods

### Generation of samples

The highly inbred ADOL chicken lines 6 (MD resistant) and 7 (MD susceptible) were inter-mated in reciprocal crosses. Half of the progeny in each cross were challenged with MDV (2,000 pfu JM strain) at 2 weeks of age. At 1, 4, 7, 11, 13, and 15 days post-infection (dpi), 12 birds per treatment group were terminated; pure line birds were similarly treated with 14 birds per treatment group. RNA from spleen was isolated and quantified.

### RNA sequencing and analysis

Two pools containing equal amounts of RNA from 6 birds each per treatment group at 4 dpi were generated, then sequenced using an Illumina GA. Using the Maq algorithm [1] to align the reads and a custom Python script, SNPs were detected, then filtered for sequence quality and alignment depth. Allelic imbalance was determined using a chi-square test (p<0.05).

### Validation using Illumina GoldenGate assays and analysis

Based primarily on Illumina design scores, assays for 1536 selected cSNPs were developed and used on RNA samples of all F_1_ birds. In addition, artificial F_1_ pools generated by mixing equal amounts of RNA from each pure line were screened to query for the influence of trans-acting factors; 12 line 6x7 F_1_ and 12 line 7x6 F_1_ DNAs were also measured to normalize the assays. The dataset was analyzed using linear models.

### Relative influence of cis- and trans-acting factors

True vs. artificial F_1_ expression ratios were plotted for each time point and condition for splenic RNAs. The total percentage of each SNP effect was estimated and the averages plotted over the course of infection.

### Pathways analysis

Biological processes were screened by inputting genes exhibiting ASE in response to MDV infection into the Metacore (GeneGo) database.

## Results and discussion

RNAs from parental lines or F_1_ progeny were obtained to evaluate potential influence of (1) maternal or epigenetic effects via reciprocal matings, (2) MDV infection, (3) time after infection, (4) different tissues, and (5) trans-acting factors via artificial F_1_ pools. As a result, we had 288 F_1_ samples and 168 artificial F_1_ RNA pools.

For ASE assays, it is necessary to have a cSNP for each allele within each gene; the polymorphism does not have to be nonsynonymous and can be in the 3’ UTR. Thus, to get a genome-wide and unbiased survey of all the expressed genes and an indication of ASE, replicate RNA pools from a single time point (4 dpi) were sequenced using a next generation sequencer. This resulted in 11-13+ million mappable reads per treatment group, 1.7+ Gb surveyed, and after filtering, 22,655 high-confidence cSNPs. Analysis of these cSNPs revealed that 5360 cSNPs in 3773 genes exhibited statistically significant allelic imbalance.

To economically validate and extend the results, 1536 cSNPs exhibiting ASE were screened on all the RNA samples of all F_1_ birds using Illumina GoldenGate arrays. In addition, artificial F_1_ pools generated by mixing equal amounts of RNA from each pure line were screened to query for the influence of trans-acting factors. The initial statistical analysis of 1465 functioning assays for ASE effects is shown in Table 1.

**Table 1:** Number of SNPs with significant sources of variation affect ASE

Source of variation	No. of significant SNPs
Direction of cross (CROSS)	481
Infection (INF)	1040
Days post infection (DPI)	1417
INF x DPI	1407
INF x CROSS	14
DPI x CROSS	1284
INF x DPI x CROSS	652
No effects	19

Besides finding genes with cis-regulatory element, the relative influence of trans-acting factors for each gene can be quantified by plotting the relative expression of each allele in true vs. artificial F_1_ RNAs as described by Wittkopp *et al.*[2]. Furthermore, the data can be further analyzed like microarrays to identify pathways that are enriched for ASE. Using the Metacore (GeneGo) database, T cell activation, vesicle mediated transport, and cell cycling were identified.

## Conclusions

Using our protocol of RNA seq followed by confirmation using Illumina GoldenGate arrays, we have identified a large number of genes exhibiting ASE in response to MDV infection. Furthermore, the direction of the cross also influences ASE, which suggests maternal or epigenetic influences. Undoubtedly, many of these genes contribute to genetic resistance to MD, which can be directly monitored in resource populations using the same cSNPs.

The described method, although demonstrated in inbred chicken lines, is applicable to all traits in any diploid species, and should prove to be a simple method to identify the majority of genes controlling any complex trait.

## Competing interests

The authors declare that they have no competing interests.

## Authors' contributions

SM carried out the sequence analyses, and participated in its design and coordination. WMM carried out the statistical and pathway analyses. SC carried out the Illumina GoldenGate assays. HHC conceived of the study, participated in its design and coordination, and helped to draft the manuscript. All authors read and approved the final manuscript.
